# Accelerometer-based measures in Friedreich ataxia: a longitudinal study on real-life activity

**DOI:** 10.3389/fphar.2024.1342965

**Published:** 2024-03-19

**Authors:** Mario Fichera, Lorenzo Nanetti, Alessia Monelli, Anna Castaldo, Gloria Marchini, Marianna Neri, Xhuljano Vukaj, Mauro Marzorati, Simone Porcelli, Caterina Mariotti

**Affiliations:** ^1^ Unit of Medical Genetics and Neurogenetics, Fondazione IRCCS Istituto Neurologico Carlo Besta, Milan, Italy; ^2^ Department of Molecular Medicine, University of Pavia, Pavia, Italy; ^3^ Institute of Biomedical Technologies, National Research Council, Segrate, Italy

**Keywords:** Friedreich ataxia, wearable sensors, activity monitor, digital measure, outcome measures

## Abstract

Quantitative measurement of physical activity may complement neurological evaluation and provide valuable information on patients’ daily life. We evaluated longitudinal changes of physical activity in patients with Friedreich ataxia (FRDA) using remote monitoring with wearable sensors. We performed an observational study in 26 adult patients with FRDA and 13 age-sex matched healthy controls (CTR). Participants were asked to wear two wearable sensors, at non-dominant wrist and at waist, for 7 days during waking hours. Evaluations were performed at baseline and at 1-year follow-up. We analysed the percentage of time spent in sedentary or physical activities, the Vector Magnitude on the 3 axes (VM3), and average number of steps/min. Study participants were also evaluated with ataxia clinical scales and functional tests for upper limbs dexterity and walking capability. Baseline data showed that patients had an overall reduced level of physical activity as compared to CTR. Accelerometer-based measures were highly correlated with clinical scales and disease duration in FRDA. Significantly changes from baseline to l-year follow-up were observed in patients for the following measures: (i) VM3; (ii) percentage of sedentary and light activity, and (iii) percentage of Moderate-Vigorous Physical Activity (MVPA). Reduction in physical activity corresponded to worsening in gait score of the Scale for Assessment and Rating of Ataxia. Real-life activity monitoring is feasible and well tolerated by patients. Accelerometer-based measures can quantify disease progression in FRDA over 1 year, providing objective information about patient’s motor activities and supporting the usefulness of these data as complementary outcome measure in interventional trials.

## 1 Introduction

The development of new outcome measures with high sensitivity in capturing time-related changes may improve the design of clinical trials allowing the reduction of treatment time and population size. In the ataxia field, the use of wearable accelerometers has been employed to capture specific gait and posture characteristics that could serve as quantitative ataxia biomarkers (Supplementary Table S1). Wearable sensors have been primarily tested in cross-sectional studies conducted in laboratory or in-clinic setting to assess gait and balance characteristics in a controlled environment (Schmitz-Hübsch et al., 2016; Terayama et al., 2018; Caliandro et al., 2019; Ilg et al., 2020; Shah et al., 2021; Velázquez-Pérez et al., 2021; Kadirvelu et al., 2023). Sensors were used to analyse several features from body movements, like gait spatial-temporal parameters, body sway, velocity and angles of body movements. These studies showed that wearable sensor technologies may enable to capture specific features differentiating patients from healthy controls and measures were correlated with clinical scales (Ilg et al., 2020; Shah et al., 2021; Kadirvelu et al., 2023).

Another approach is to use wearable sensors to assess physical activity performed by the subjects during free living conditions. This type of remote monitoring allowed the collection of large sets of data with continuous activity recording for several days (Subramony et al., 2012; Milne et al., 2021; Mueller et al., 2021; Khan et al., 2022; Eklund et al., 2023; Gupta et al., 2023). Several cross-sectional studies, applying different techonolgies and protocols, collected information regarding patient’s ability to perform daily physical activity, energy expenditure, and numbers of steps. Two studies tested wearables both in-clinic and in a free-living environment (Ilg et al., 2020; Thierfelder et al., 2022). Patients were required to perform a defined in-clinic protocol and then to wear the devices in the subsequent few hours in a free-living context. The results of these studies showed a high correlation between laboratory-based and free-walking conditions, with the highest effect size observed in real-life walking (Ilg et al., 2020; Thierfelder et al., 2022).

Despite the large number of cross-sectional studies, only few longitudinal studies have been performed in patients with ataxia (Milne et al., 2021; Gupta et al., 2023). Longitudinal data are essential to support the use of wearable technologies in therapeutical clinical trials to complement traditional outcome measures in quantifying treatment effect on patient daily life.

Here we present a 12-month longitudinal study monitoring real-life physical activity in patients with Friedreich ataxia (FRDA). FRDA is an autosomal recessive multi-systemic neurodegenerative disorder that represents the most frequent hereditary ataxia in the Caucasian population. The disease is caused in >95% of cases by a repeated GAA expansion in intron 1 of the frataxin (FXN) gene that encodes for the frataxin protein. The patients usually manifest gait instability, limb incoordination and dysmetria, before the age of 25 years (Reetz et al., 2015). The first treatment for patients with FRDA, Omaveloxolone, was approved by FDA in 2023 (Subramony and Lynch, 2023), and additional other compounds and different therapeutical strategies are currently being tested. The response to treatments in clinical trials is currently measured using neurological scales, such as the Scale for the Assessment and Rating of Ataxia (SARA; Schmitz-Hübsch et al., 2006) and the Friedreich’s Ataxia Rating Scale (FARS; Rummey et al., 2019). At present, only one previous longitudinal study with wearable sensors has been performed in FRDA (Milne et al., 2021).

In our prospective study, we evaluated a population of patients with FRDA with the following aims: (i) assess feasibility of 1-week remote free-living monitoring, (ii) detect effective changes in accelerometer-based measures at 1-year interval, (iii) identify useful measures of disease progression, and (iv) evaluate possible correlations between accelerometer-derived measures and clinical scores.

## 2 Methods

### 2.1 Participant and clinical evaluations

In this study we enrolled patients with: (1) confirmed genetic diagnosis of FRDA, (2) age between 18 and 45 years, (3) onset of neurological signs before 25 years, and (4) maintained ambulatory capacity (SARA gait score <8). Age and sex matched healthy control subjects were also enrolled. Study participants were evaluated at baseline and after a 12-month interval. Demographical characteristics (age, sex, weight and height, Body Mass Index–BMI) were obtained from all subjects. All subjects underwent clinical evaluation with SARA (Schmitz-Hübsch et al., 2006) and modified FARS scales (Rummey et al., 2019), with functional tests for upper limbs dexterity (Composite Cerebellar Function Severity score - CCFS; Tezenas du Montcel et al., 2008) and walking capability (8 m walk test - 8MWT; Fahey et al., 2007). GAA expansion size on both alleles (GAA1 and GAA2) was available for all patients.

### 2.2 Activity monitoring with wearable accelerometers

To assess real-life activity, we used the triaxial accelerometer ActiGraph GT3X-BT (ActiGraph LLC, Pensacola, FL). Participants were provided with instructions on how to position the devices, and were asked to wear two ActiGraphs: one at the non-dominant wrist and the other at waist, with a belt over the L5 vertebra. Both devices were worn during waking hours for 7 consecutive days. All subjects were asked to fill-in a diary providing information regarding the exact time when the devices were worn and daily physical activities. At the end of the acquisition period, the accelerometers and diaries were returned by the patients via mail.

ActiGraphs collected data at a sampling rate of 30 Hz. The ActiLife software (version 6.13.4) was used to extract the raw accelerometer data from the ActiGraph and to calculate selected variables from raw data. Low Frequency Extension (LFE) was applied to raw data, since it has been deemed more sensitive in neurologically impaired individuals (Hicks et al., 2019). Data were summarized into 60-s intervals (epochs) and raw accelerometer files were processed to identify non-wear periods. Non-wear periods were identified using the non-wear time classification algorithm reported by Choi et al. (Choi et al., 2011), and correction were made to fit data with subjects’ log. Days with less than 8 h of total wear time were excluded from the analysis. Accelerometer data were expressed as activity counts per minute (CPM). CPM were used to define the following variables: (1) Metabolic Equivalent of Task (MET) estimates score; (2) number of activity bouts; (3) % of activity; (4) Vector magnitude (VM3) and (5) step count.

MET rate is a measure of the energy expenditure of a subject, relative to the mass of that person. One MET corresponds to the consumption of 3.5 mL of oxygen per kg of body weight per minute, and corresponds approximately to the amount of energy spent while sitting quietly.

The number of activity bouts was defined as the number of events where the subject performs moderate physical activity lasting at least 10 min (Freedson et al., 1998). Percentage of activities defines the time spent by subjects in sedentary, light, or moderate/vigorous (MVPA) activity and was calculated using published cut-offs on vertical axis (Matthews et al., 2005). VM3 expresses the mean CPM of the vector resulting from CPM on the 3 axes and is thus a raw value. Step count was defined as the average number of steps/minute, and is calculated by a proprietary algorithm of the ActiLife software.

The study was evaluated and approved by the Local Ethic Committee on 13 January 2021 (Protocol N. 80). All subjects gave written informed consent for the participation in the study, and all procedures were carried out in accordance with the Declaration of Helsinki.

### 2.3 Statistical analyses

At baseline, inter-group differences between healthy controls and subjects with FRDA were assessed using Mann–Whitney test or *t*-test according to data distribution. Spearman correlation test was used to correlate activity data with clinical and demographical variables. Paired sample *t*-test or Wilcoxon ranked sign test was used to assess longitudinal differences. Data are presented as mean ± standard deviation (SD). Bonferroni correction was applied for multiple comparisons of unrelated parameters. In a preliminary analysis we checked and confirmed that measures extracted from wrist and waist devices were highly correlated in both FRDA and controls, thus we corrected for total number of activity parameters without considering wrist and waist as independent measures. Test-retest reliability of digital measures was assessed using Intraclass Correlation Coefficient (ICC). ICCs were used to estimate minimal detectable change (MDC), defined as the minimal threshold beyond the random measurement error with a 95% confidence level (Ilg et al., 2023).

Reponsiveness of clinical and digital measures was assessed using standardized response mean (SRM), calculated as (mean change)/(standard deviation of the change). SRM larger than 0.8 indicates large responsiveness, 0.5–0.8 moderate, and <0.5 low. SRM was used to determine sample sizes of a two-arms clinical trial using a 2-tailed type I error of <5%, a power of 80%, and 50% reduction in disease progression (Milne et al., 2021; Ilg et al., 2023).

The level of significance was set at *p* < 0.05. Statistical analyses were conducted using JMP^®^, version 11 (SAS Institute Inc., United States).

The data that support the findings of this study are available from Open Repository of the Fondazione IRCCS Istituto Neurologico Carlo Besta upon reasonable request to the corresponding author (https://zenodo.org/communities/besta). The data are not publicly available as they contain information that could compromise the privacy of research participants.

## 3 Results

### 3.1 Participants

Between October 2021 and July 2022, we enrolled 26 subjects with FRDA and 13 healthy controls. FRDA patients (16M/10F) had a mean age of 27.1 ± 7.8 years (range 18–43), age at onset was 15.4 ± 4.9 years (range 7–24) and mean disease duration was 11.7 ± 6.9 years (range 1–28). SARA score was 17.2 ± 6.0 points, mFARS score was 50.5 ± 13.7 points, and ADL score was 12.3 ± 5.5 points. Control subjects, 7 women and 6 men, had a mean age of 25.9 ± 3.1 years (range 21–31), and the scores at clinical scales ranged between 0 and 1 point.Patients had a mean CCFS score of 1,225 ± 124 points (controls = 947 ± 282), and 13 out of 26 patients were able to complete the 8MWT, performing the task in 8.7 ± 3.9 s (controls = 4.2 ± 0.6 s).

Between September 2022 and June 2023, 21 patients with FRDA (81%) and 11 Control subjects (85%) returned to our clinical center to perform 1-year follow-up examinations (Table 1). Mean time interval between baseline and follow-up was 11.6 ± 1.6 months. In comparison with baseline evaluations, patients with FRDA had a mild worsening of ataxia signs with a mean increase of 1.5 ± 1.5 points in SARA score (*p* < 0.01), 0.8 ± 4.6 points in mFARS score (p = *n*.s), and 1.0 ± 2.4 points in ADL score (p = *n*.s.). The scores obtained at both CCSF and 8MWT functional measures did not differed between baseline and follow-up. In controls no differences in clinical and functional measures were observed (Table 1).

**TABLE 1:** Longitudinal clinical scores and digital measures in patients with Friedreich ataxia and healthy controls.

	Controls			FRDA			
	Baseline	Follow-up	*p*-value	Baseline		Follow-up	*p*-value*
**N**	10	10		20	20		
**SARA**	0	0	-	17.5 ± 6.1	19.0 ± 6.2		<0.01
**mFARS**	1.0 ± 1.4	0.0 ± 0.0	-	51.3 ± 14.0	52.1 ± 12.5		-
**ADL**	0	0	-	13.1 ± 5.1	14.1 ± 4.6		-
**CCFS**	962.8 ± 309.2	847.4 ± 49.0	-	1,233.6 ± 128.6	1,240.1 ± 112.2		-
**8MWT** ^**#**^	4.2 ± 0.7	5.7 ± 0.7	-	7.5 ± 2.5	8.1 ± 2.2		-
**Metabolic Equivalent of task rate**							
***wais***	1.19 ± 0.09	1.18 ± 0.09	-	1.02 ± 0.02	1.01 ± 0.01		-
***wrist***	1.65 ± 0.16	1.61 ± 0.14	-	1.37 ± 0.27	1.33 ± 0.24		-
**N. of activity bouts**							
***Waist***	8.8 ± 4.4	6.5 ± 5.8	-	0.2 ± 0.9	0.0 ± 0.0		-
***Wrist***	48.7 ± 16.3	36.4 ± 13.1	-	26.7 ± 30.5	19.1 ± 23.5		-
**Sedentary activity (%)**							
***Waist***	67.7 ± 6.7	69.0 ± 4.9	-	81.3 ± 9.4	84.1 ± 7.9		<0.01
***wrist***	31.7 ± 8.1	32.2 ± 8.2	-	42.9 ± 13.0	45.8 ± 13.1		-
**Light activity (%)**							
***Waist***	21.8 ± 5.3	20.2 ± 5.0	-	16.0 ± 7.4	13.8 ± 6.6		<0.01
***wrist***	24.9 ± 3.1	25.8 ± 5.8	-	25.8 ± 5.0	26.7 ± 4.6		-
**Moderate-Vigorous activity (%)**							
***Waist***	10.5 ± 3.2	10.8 ± 2.6	-	2.7 ± 2.4	2.1 ± 1.7		-
***wrist***	43.4 ± 8.9	42.0 ± 7.0	-	31.3 ± 13.3	28.2 ± 12.1		0.01
**Vector Magnitude (CPM)**							
***Waist***	563.2 ± 159.0	535.2 ± 15.7	-	277.0 ± 155.3	228.8 ± 126.5		<0.01
***wrist***	2387.3 ± 474.7	2285.8 ± 333.3	-	1,656.5 ± 758.9	1,517.0 ± 679.1		-
**Steps/minute**							
***Waist***	16.0 ± 3.6	15.7 ± 1.9	-	7.7 ± 4.1	6.5 ± 3.4		-
***wrist***	20.2 ± 3.5	18.4 ± 5.6	-	14.0 ± 5.1	12.6 ± 4.9		-

*p*-value after Bonferroni-corrected paired Wilcoxon test between baseline and follow-up; only *p* < 0.05 are reported. Data are mean ± SD; SARA: Scale for the Assessment and Rating of Ataxia. mFARS: modified Friedreich Ataxia Rating Scale; ADL: Activity of Daily Living part of FARS; CCFS: cerebellar composite functional severity score; CPM: counts per minute.

b8MWT: 8 m walk test (seconds), was performed at follow-up by 9 patients.

### 3.2 Activity monitoring with wearable accelerometers

All subjects completed 1-week remote assessment with good adherence to the established protocol. Only one subject with FRDA lost the wrist-worn Actigraph during the baseline assessment period. The number of days with viable accelerometer data was 6.92 in FRDA patients and 6.84 in control subjects.

At 1-year follow-up, the mean number of days with viable data was 6.95 in FRDA and 6.54 in Controls. One patient with FRDA and one control subject wore the devices only for 3 and 2 days respectively, and were excluded from analyses.

To assess test-retest reliability of Actigraph measurements, we compared odd weekdays (Monday-Wednesday-Friday) *versus* even weekdays (Tuesday-Thursday-Saturday), recorded at baseline. Intraclass Correlation Coefficients (ICC) indicate good reliability of the measures, for sedentary activity at waist (coefficient0.79), VM3 both at waist (0.82) and at wrist (0.86), and for MET rate (0.89), number of activity bouts (0.83 (Supplementary Table S1; Supplementary Figure S1). Similar results were also obtained for follow-up data.

#### 3.2.1 Baseline data

**FIGURE 1:**
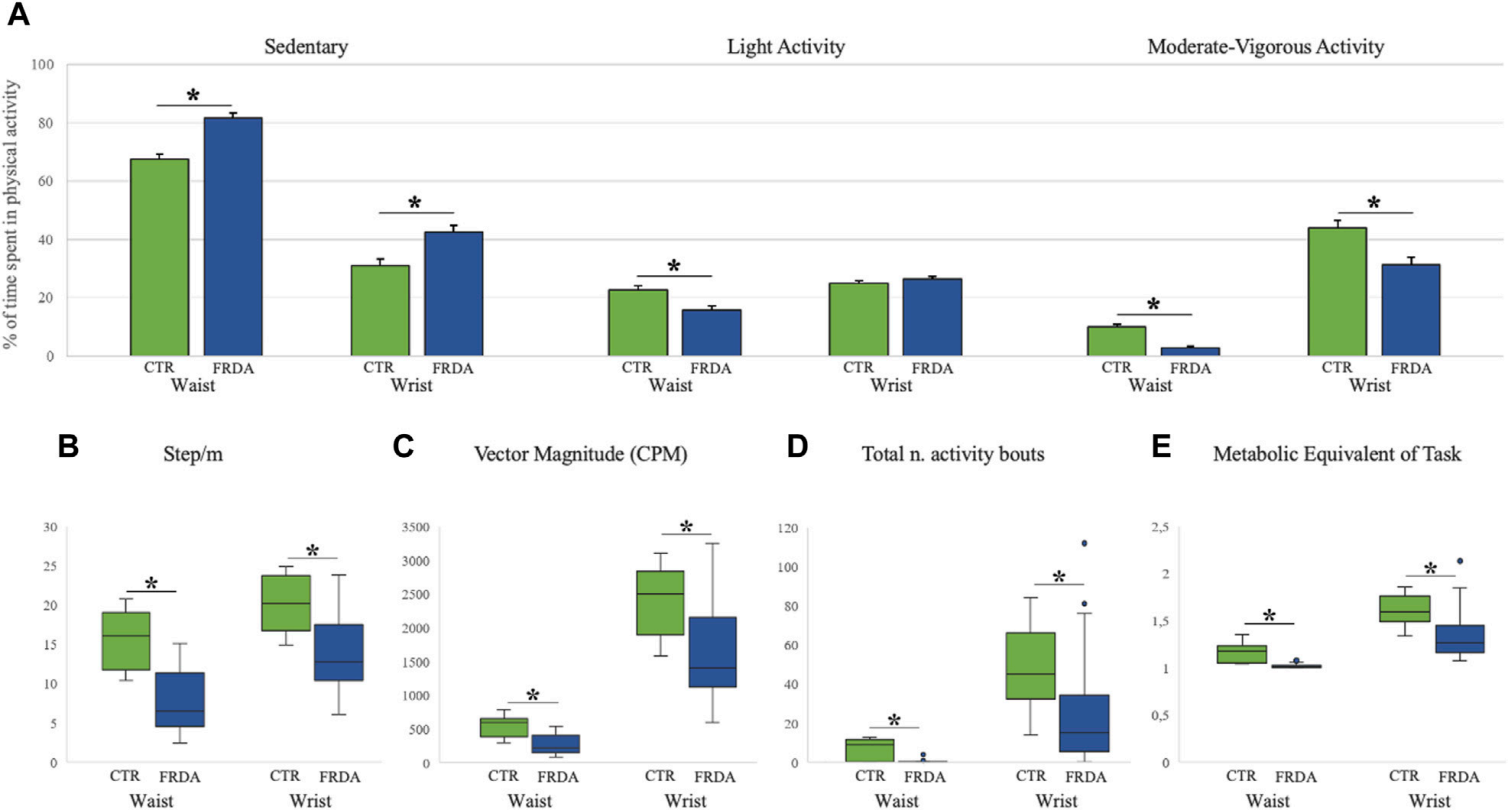
Baseline Actigraph measures in patients with Friedreich ataxia and in healthy controls Measures of physical activity in patients with Friedreich ataxia (FRDA, blue columns) and in controls (CTR, green columns) are reported for both wrist- and waist-worn sensors. Panel **(A)** shows the percentages of sedentary, light and moderate-vigorous activities (mean, standard error). Panels **(B–E)** display box and whisker plots of accelerometer data for: Step/minute [graph **(B)**]; Vector Magnitude over 3 axes [graph **(C)**]; total number of activity bouts [graph **(D)**]; and metabolic equivalent of task rate [graph **(E)**]. Boxes show median value (middle line) and inter-quartile range (25%–75%), whiskers extend to upper and lower quartiles, dots represent outliers. *p < 0.05 for comparison between FRDA and controls (after Bonferroni correction).

Baseline data of activity monitoring showed that FRDA patients had an overall reduced level of physical activity as compared to control subjects. For both FRDA and control subjects, the physical activity recorded at wrist gave higher values than the activity recorded at waist for all accelerometer measures. Patients showed a higher percentage of time spent in sedentary activity recorded both at waist (82%) and at writs-worn (42%) accelerometers in comparison with controls (67% and 31%), and a much less frequent MVPA activity (3% at waist and 31% at wrist) in comparison with controls (10% and 44%). On the contrary, the percentage of light activity was similar in patients and in controls (Figure 1A). Significant differences between patients and controls were also observed for other measures of daily activities, such as the average numbers of steps (Figure 1B), the vector magnitude of movements (Figure 1C), the total number of activity bouts (Figure 1D), and energy expenditure (Figure 1E). At baseline, patients who completed the 8MWT (N = 13) had shorter disease duration, later age at onset and spent less time in sedentary activity compared with patients unable to complete the walking test (N = 13) (Supplementary Table S2).

#### 3.2.2 Longitudinal data

In patients with FRDA all variables indicated a reduced physical activity level during daily life compared to baseline assessments, while no changes were identified in controls (Table 1).

**FIGURE 2:**
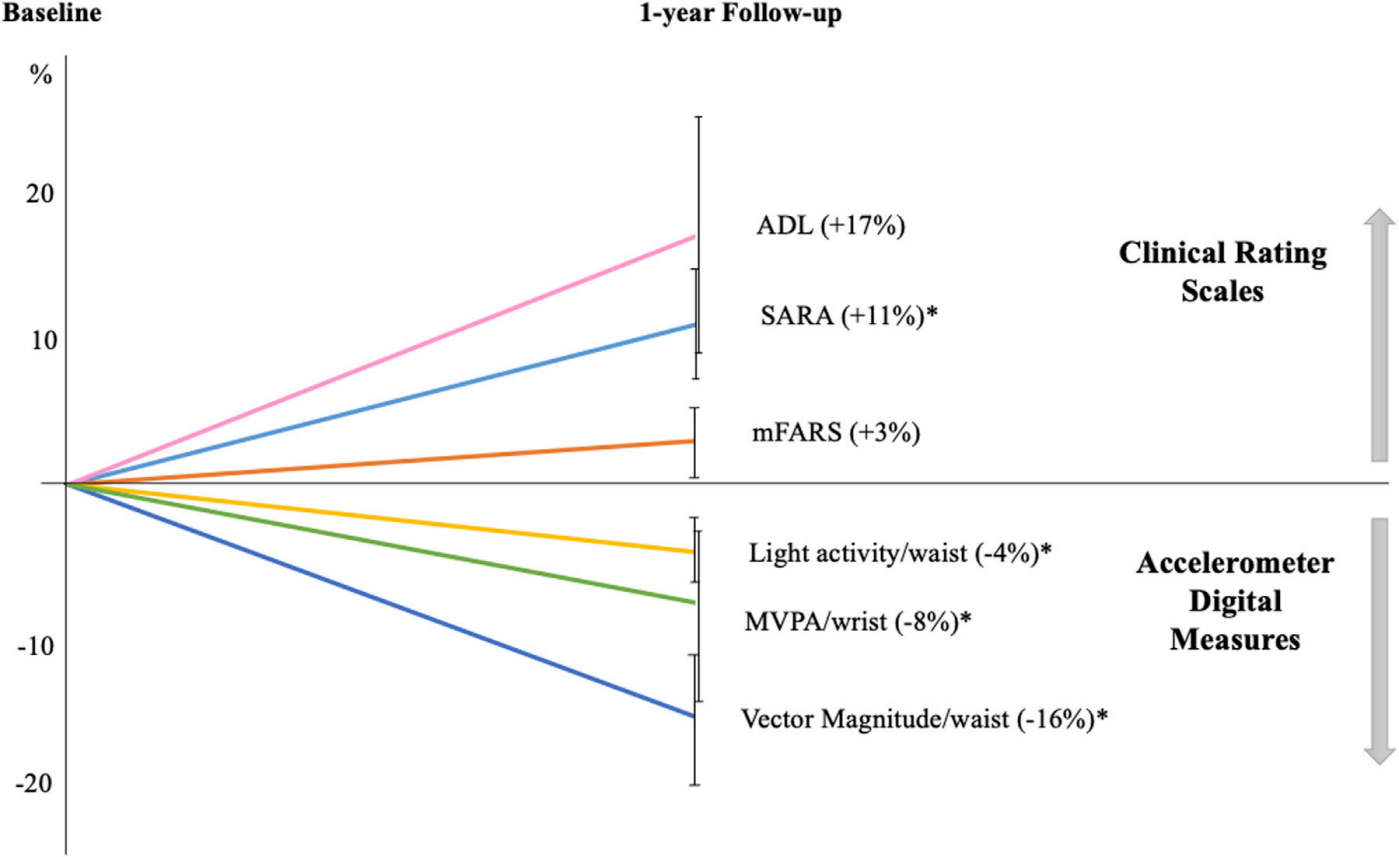
One-year longitudinal changes in Friedreich patients Graph displays the percentages of variations in clinical and digital measures at follow-up compared to baseline. All clinical scale scores increased at follow-up indicating a worsening of ataxia severity: SARA (Scale for the Assessment and Rating of Ataxia); mFARS (modified Friedreich Ataxia Rating Scale); and ADL (Activity of Daily Living part II of FARS scale). Accelerometer digital measures showed a reduction in daily-life motor activities, as indicated by the decreased percentage of light and moderate-vigorous activities (MVPA) and in vector magnitude. Error bars indicate standard error; *p < 0.05 at paired-statistical test comparing follow-up and baseline data for.

After correction for multiple comparisons, the measures that significantly changed from baseline to l-year follow-up were: (i) VM3; (ii) the percentage of sedentary and light activity recorded by waist sensor, and (iii) the percentage of MVPA at wrist sensor. More in detail, VM3 was 277.0 ± 155.3 CPM at baseline and 228.8 ± 126.5 at follow-up, with a mean decrease of approximately 16% (*p* < 0.01). Sedentary time was 81.3% ± 9.4% at baseline and increase to 84.1% ± 7.9% at follow-up (4% increase; *p* < 0.01) (Figure 2). Patients with FRDA showed reduced time spent in both light physical activities (13.8% ± 6.6% vs 16.0% ± 7.4%, at waist sensor) and moderate-vigorous activities (28.2% ± 12.1% vs 31.3% ± 13.3%, at wrist sensor) (Figure 2).

At 1-year follow-up, subjects unable to complete the 8MWT showed no significant changes in physical activity, except for a reduced number of activity bouts recorded from wrist sensor (*p* = 0.05, Bonferroni uncorrected). The patients that at baseline were able to perform the test, showed a reduction in Vector Magnitude (*p* = 0.014), in the number of steps/minute (*p* = 0.022), and an increase in sedentary activity (*p* = 0.007) at follow-up.Two of 10 subjects were unable to perform the 8MWT.

### 3.3 Correlations

**FIGURE 3:**
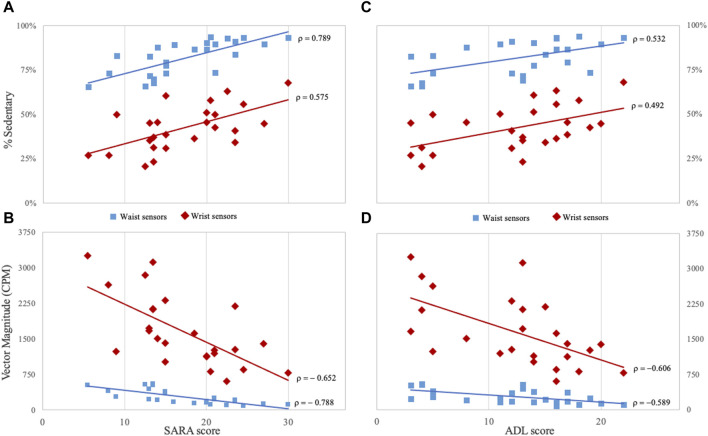
Correlations between clinical and digital measures in Friedreich patients **(A, B)** panels show the correlation between SARA score and the percentage of time spent in sedentary activities and Vector Magnitude. **(C, D)** panels show the correlation between ADL score and the same digital measures. Measures derived from for waist sensors are indicated by light-blue squared dots, and measures from wrist sensors by red rhombuses. CPM: counts per minute; SARA: Scale for the Assessment and Rating of Ataxia; ADL: Activity of Daily Living part II of Friedreich Ataxia Rating Scale. All correlations are statistically significant (*p* < 0.05) after Bonferroni correction for multiple comparisons.

At baseline, the majority of digital measures derived from Actigraph sensors correlated with scores of the clinical rating scales (SARA, mFARS and ADL) (Figure 3). Years of disease duration correlated with waist-derived measures of sedentary activity (ρ = 0.502); VM3 (ρ = −0.590); and step/min (ρ = −0.524).

Sedentary activity, VM3, and Step/m registered at waist, also showed the highest correlation coefficients with SARA scores (ρ = 0.789, ρ = −0.788 and ρ = −0.786, respectively), and with mFARS scores (ρ = 0.748, ρ = −0.726 and ρ = −0.714, respectively) (Figure 3). ADL scores were also correlated with sedentary activity (ρ = 0.532) and VM3 recorded at waist (ρ = −0.589) (Figure 3).

CCFS score was correlated with sedentary activity (ρ = 0.675), VM3 (ρ = −0.748) and Step/m (−0.728) from waist sensor, and with the number of activity bouts (−0.597) and Step/m (−0.594) detected from wrist sensor. No correlation between 8MWT and digital measures was observed. We examined the correlation between accelerometer measures and FARS subscales. FARS part E (upright stability) was correlated with sedentary time (waist, ρ = 0.781), number of activity bouts from wrist sensor (ρ = −0.670), VM3 from waist (ρ = −0.742) and wrist (ρ = −0.636), and step/m from waist (ρ = −0.771) and wrist (ρ = −0.644). Only a few correlations were identified for mFARS subscore for upper and lower limbs, and no significant correlations were identified between activity data and FARS part A (bulbar) (Supplementary Table S3).

At 1-year-follow-up evaluations, we confirmed the correlations observed at baseline. Longitudinal changes [(follow-up measure) - (baseline measure)] in digital measures did not correlate with longitudinal changes in SARA total score. Only the changes in SARA “gait” sub-score correlated with changes at waist-VM3 (ρ = −0.526; *p* = 0.02) and at sedentary activity (ρ = 0.517; *p* = 0.03; Bonferroni uncorrected).

Based on the effect size, we estimated the sample population number for a future two-arm interventional trial, considering both activity measures and clinical scores. The lowest sample sizes were obtained for SARA score (N = 22); waist-VM3 (N = 30); and % of sedentary activity at waist (N = 38) (Table 2).

**TABLE 2:** Effect size coefficient and estimated sample size for clinical and Actigraph variables.

	SRM	Sample size per treatment arm
SARA score	0.98	22
mFARS score	0.16	618
ADL	0.39	108
CCFS	0.09	1940
8MWT	0.29	194
Metabolic Equivalent of Task rate		
*Waist*	0.33	150
*wrist*	0.34	140
N. of activity bouts		
*Waist*	0.27	220
*wrist*	0.43	90
% sedentary		
*Waist*	0.69	38
*wrist*	0.36	126
% Moderate-Vigorous physical activity		
*Waist*	0.59	50
*wrist*	0.51	66
VM3 (CPM)		
*Waist*	0.79	30
*wrist*	0.46	80
Steps/minute		
*Waist*	0.54	58
*wrist*	0.51	66

Sample size per treatment arm is calculated for 1-year study comparing two treatment groups, with 80% power and α = 0.05. SRM: standardized response mean; SARA: Scale for the Assessment and Rating of Ataxia. mFARS: modified Friedreich Ataxia Rating Scale; ADL: Activity of Daily Living part of FARS; CCFS: cerebellar composite functional severity score; 8MWT: 8 m walk test (seconds); n.a.: not assessed; VM3 CPM: vector magnitude counts per minute.

## 4 Discussion

Disorders of the cerebellum cause several neurological motor signs such as disequilibrium, limb incoordination and speech difficulties. The severity of motor impairment is currently evaluated and graded by the scores obtained using specific clinical scales or functional tests measuring walking speed or manual abilities. Recently several devices and new tools have been developed to obtain quantitative measures that could improve the accuracy of the scales and tests performed in a clinical setting during the neurological examination, and allow the monitor of a larger time frame of the patient real life (Minen and Stieglitz, 2021). Some of these assessments may be performed remotely and digital devices may record the patient activities in a home environment. These new tools are of great interest for establishing the impact of potential therapeutic intervention of the real-life activity of patients with movement disorders (Lipsmeier et al., 2022). To assess the use of accelerometer devices as effective outcome measures in interventional trials it is important to evaluate feasibility and reliability of activity measurements in tracing longitudinal changes during disease progression.

Remote real-life physical monitoring has been previously assessed in ataxic patients both in FRDA (Mueller et al., 2021) and in other types of ataxias (Khan et al., 2022; Eklund et al., 2023; Gupta et al., 2023) (Supplementary Table S1). Mueller et al. tested different home-based digital endpoints evaluating speech, hand function and gait (Mueller et al., 2021) in a cross-sectional study *versus* controls. Several parameters discriminated FRDA patients from controls, including activity monitoring parameters recorded from feet and wrist sensors, but not from trunk (Mueller et al., 2021). In a second study, Milne et al. performed a 1-year longitudinal study including measures assessing gait, balance and remote activity monitoring. A significant change in activity duration, daily step count and distance walked compared to baseline was observed after 6 months, while significant decline was present only in daily step count and distance walked after 12 months (Milne et al., 2021).

We decided to analyse very simple accelerometer-derived measures: the overall level of physical activity, from sedentary to vigorous activity, the VM3 indicating the total amount of movement in the three-dimensional space (3 axes), and the step counts per minute. Each study participant worn two Actigraph sensors, one at waist and one at wrist of the non-dominant hand. As expected, overall activity monitoring showed less and lighter movement activities in subjects with FRDA than in healthy controls. Patients were significantly more sedentary than controls, and were also less likely to perform sustained physical activities (i.e., activity bouts) compared to controls. Only light activity calculated from wrist sensors was similar in the two groups of participants. While differences in activity measures between patients and controls were expected, our major interest was to observe longitudinal data in the patient group, and test the hypothesis that real-life activity could reliably capture changes related to disease progression in 1-year interval.

We observed greater levels of activity at wrist-recorded sensors in comparison with waist sensors. In a previous study, the same observation in ataxic patients has been interpreted as an overestimation of activity related to patient’s upper limb dysmetria and tremor (Mueller et al., 2021). In the present study, the increased movement activity at upper-limb was not disease-specific but was accounted in both patient and control participants, suggesting a physiological occurrence of more activity at upper limbs than at the trunk level.

Activity monitoring in ataxic patients has been tested with different type of sensors that have been have been placed at dominant wrist (Khan et al., 2022; Gupta et al., 2023), ankle (Subramony et al., 2012), dominant wrist and ankle (Eklund et al., 2023), triceps (Milne et al., 2021), feet, trunk and wrists (Mueller et al., 2021) (Supplementary Table S4). Some authors have compared the performances of sensors according to placement, with different results. Mueller and co-authors (Mueller et al., 2021) found that trunk sensor did not discriminate between FRDA patients and controls, while differences between disease and control groups were better captured using feet and wrist-worn sensors.

Digital measures were significantly related with ataxia rating scales (Subramony et al., 2012; Mueller et al., 2021; Gupta et al., 2023) and with patient perception of disability measured via a patient reported questionnaire (Eklund et al., 2023). We confirm that activity measures in FRDA are highly correlated with disease duration, clinical scales, and functional tests, while are not influenced by demographic variables, such as age, sex and BMI.

At 1-year interval, all sensor-derived measures (both from waist- and wrist-worn sensors) showed a trend toward reduced activity in patients, and remained unchanged in controls. After correction for multiple comparisons, we demonstrated a significant effect of time in VM3, sedentary and light activity time (waist) and MVPA (wrist). VM3 measures had lower variability compared to the other activity measures, and were associated with the highest effect size. Milne and colleagues described a reduced activity in terms of physical activity duration, step count and distance walked in FRDA patients after a 6-month interval. However, the same authors reported that at 12 months-follow-up only step count continued to show a progressive decline, suggesting that clinical assessment with FARS outclassed digital measures in the sensitivity to longitudinal changes (Milne et al., 2021). Gupta and colleagues (Gupta et al., 2023), conducted a longitudinal study in patients with Ataxia-telangiectasia (A-T) showing reduced activity indexes after a 1-year interval. These authors provided a novel approach in the analyses of the data allowing the extraction of characteristic movements from wrist sensor and identifying several sub-movement features that distinguished patients from controls. Although the recorded movements were highly disease specific, their measures showed no substantial changes at 1-year interval.

In our study we assessed physical activity during daily life, not limited to a specific task or setting. Our results suggest that the reduction in physical activity observed at follow-up in FRDA patients is mostly related to a worsening in gait performances, as showed by the presence of significant correlation of the change in SARA gait sub-score with VM3 and sedentary time. A longitudinal study in amyotrophic lateral sclerosis, that adopted the same monitoring setting of our study, showed that VM3 was the most reliable digital indicator of functional progression (van Eijk et al., 2019). In our study, we confirmed that VM3 measure represent the most valuable digital measure for future clinical trials, as showed by the highest effect size (Table 2).

As new treatments are becoming available for FRDA (Subramony and Lynch, 2023), it is crucial to link the improvement observed during clinical assessments with the improvements in patients’ ability to perform daily activities. The activity measures presented here, such as the time spent in different levels of physical activities and step count, could represent sensitive measures linked to day-to-day functioning of patients. Importantly, we used waist- and wrist-worn sensors to collect data from ambulatory patients, however the same protocol could also apply to patients with reduced or absent ambulatory capacity.

Using wearable devices during real-life activities comes with some limitations. Due to the free-living context where these data were collected, the exact nature of the activities recorded is unknown. Moreover, the analysis of activity was based on proprietary software (Actilife) that lacks an algorithm specific for the ataxic population, over- or under-estimating performances and introducing other possible bias. Due to the limited number of subjects, the present work has to be considered as a pilot exploratory study. Possibly a larger number population size would be needed to prove that the observed longitudinal changes are above measurement noise (Supplementary Table S1). Our data, however, suggest that VM3 could represent a viable solution to assess total level of physical activity, as it represents the raw value of the vector recorded by the devices, and does not introduce bias related to post-processing.

In conclusion, our results indicate that activity monitoring using Actigraph devices represent a well-tolerated and sensitive tool for individuals with FRDA. Based on the longitudinal data and the preliminary results on the test-retest-reliability, the waist sensors seem to be more reliable than wrist sensors. In addition waist sensor measures showed good correlations with. clinical scale and daily life activity rating scores, supporting the usefulness of waist-based accelerometer to measure activity in future clinical trials in FRDA.

## Data Availability

The datasets presented in this study can be found in online repositories. The names of the repository/repositories and accession number(s) can be found below: Open Repository (OR) of Fondazione IRCCS Istituto Neurologico Carlo Besta (https://zenodo.org/communities/besta).
