# Supplementary material for: Accelerometer-based measures in Friedreich ataxia: a longitudinal study on real-life activity
Source: Front Pharmacol. 2024 Mar 19;15:1342965. doi: 10.3389/fphar.2024.1342965 (PMC10985256; doi:10.3389/fphar.2024.1342965)
Supplement: Supplementary file 2 [file DataSheet1.docx]

**Supplementary Material**

**Accelerometer-based measures in Friedreich ataxia:**

**a longitudinal study on real-life activity**

Fichera M, Nanetti L, Mongelli A, Castaldo A, Marchini G, Neri M,

Vukaj X, Marzorati M, Porcelli S, Mariotti C.

**Supplementary Table 1.**

**Test-retest analyses and Minimal Detectable Change**

|  | Waist | | | Wrist | | |
| --- | --- | --- | --- | --- | --- | --- |
|  | ICC | SEM | MDC_95%_ | ICC | SEM | MDC_95%_ |
| Metabolic Equivalent of Task | 0.38 | 0.015 | 0.041 | 0.89 | 0.089 | 0.245 |
| N. of activity bouts | n.a. | n.a. | n.a. | 0.83 | 2.06 | 5.70 |
| % sedentary | 0.79 | 4.80 | 4.81 | 0.70 | 7.48 | 20.74 |
| Vector Magnitude (CPM) | 0.82 | 68.97 | 68.96 | 0.86 | 292.95 | 812.00 |
| Step/minute | 0.70 | 2.49 | 6.89 | 0.77 | 2.42 | 6.70 |

Test-retest reliability coefficient evaluated with Intraclass Correlation Coefficient (ICC) between odd and even weekdays in FRDA patients for Actigraph variables at baseline. N.a.: not assessed; CPM: counts per minute. SEM: Standard Error of Measurements; MDC_95%_: Minimal Detectable Change at 95% confidence.

**Supplementary Figure 1. Bland-Altmann Plots for test -retest analyses**

**Supplementary Table 2. Comparison between patients able or unable to perform the walking test**

|  | Able  to complete  8MWT | Unable  to complete  8MWT | p-value (uncorrected) |
| --- | --- | --- | --- |
| N. Patients | 13 | 13 |  |
| N. patients using walking aid (walker) | 2 | 10 |  |
| SARA | 12.6±3.3 | 21.8±12.6 | <0.001 |
| Age at onset | 17.3±4.1 | 13.5±4.9 | 0.047 |
| Disease duration | 9.6±6.0 | 14.7±5.1 | 0.006 |
| MET  waist  wrist | 1.03±0.02  1.47±0.28 | 1.01±0.01  1.21±0.12 | 0.018  0.006 |
| N. of activity bouts  waist  wrist | 0.4±1.1  42.4±32.1 | 0.0±0.0  7.8±7.9 | n.a.  <0.001 |
| % sedentary  waist  wrist | 74.9±7.8  36.0±11.5 | 88.2±6.0  49.3±10.2 | <0.001  0.008 |
| VM3  waist  wrist | 386.1±142.1  2085.2±711.9 | 168.1±76.5  1163.6±412.6 | <0.001  0.002 |
| Step/m  waist  wrist | 10.3±3.5  16.4±4.9 | 5.0±2.7  11.1±3.2 | <0.001  0.011 |

Baseline characteristics of FRDA patients able and unable to complete the 8-meter walking test (8MWT). Data are shown as mean±s.d. MET: Metabolic Equivalent of Task; VM3: Vector Magnitude over 3 axes, counts-per-minute; n.a.: not assessed.

**Supplementary Table 3. Correlation between FARS sub-scores and activity measures**

| **FARS subscale** | **MET rate** | **N. of activity bouts** | **% sedentary activity** | **Vector Magnitude**  **CPM** | **Step/m** |
| --- | --- | --- | --- | --- | --- |
| **Part A**  **waist**  **wrist** | n.s.  n.s. | n.s.  n.s. | n.s.  n.s. | n.s.  n.s. | n.s.  n.s. |
| **Part B**  **waist**  **wrist** | n.s.  n.s. | n.s.  n.s. | 0.614  n.s. | -0.592  -0.581 | n.s.  n.s. |
| **Part C**  **waist**  **wrist** | n.s.  n.s. | n.s.  -0.634 | 0.634  n.s. | n.s.  -0.605 | -0.591  n.s. |
| **Part D**  **waist**  **wrist** | n.s.  n.s. | n.s.  -0.645 | n.s.  -0.706 | n.s.  -0.692 | n.s.  n.s. |
| **Part E**  **waist**  **wrist** | n.s.  n.s. | n.s.  -0.670 | 0.781  n.s. | -0.742  -0.636 | -0.771  -0.644 |

Correlation coefficient between Friedreich Ataxia Rating Scale (FARS) sub-scores and activity measures. FARS part A: bulbar; part B: upper limbs; part C: lower limbs; part D: peripheral nervous system; part E: upright stability. Coefficients are shown only for statistically significant correlations after Bonferroni correction for multiple comparisons. MET: Metabolic Equivalent of Task; CPM: counts per minute; n.s.: not significant.

**Supplementary Figure 2.**

**Correlation between longitudinal changes in SARA score and Vector Magnitude**


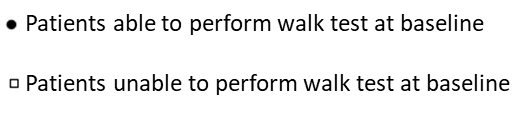
**
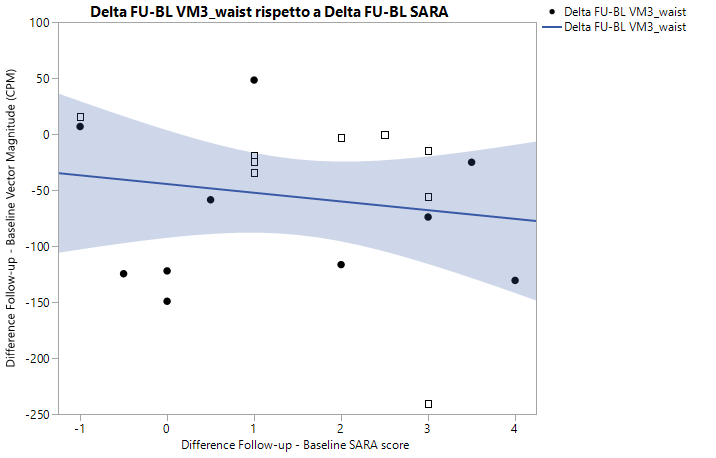
**

**Figure 1.** Scatter plot showing the correlation between longitudinal changes in SARA score and Vector Magnitude for waist sensor in FRDA patients. The correlation is not statistically significant. CPM: counts per minute.

**Suppl. Figure 3A-2B.** Correlations between longitudinal changes in SARA gait score and activity measures Vector Magnitude for waist sensor in FRDA patients.

Scatter plot showing the correlation between longitudinal changes in SARA gait score and Vector Magnitude for waist sensor in FRDA patients (**Figure 2A)**. The correlation is statistically significant (ρ= -0.526; p=0.02, Bonferroni uncorrected).  **Figure 2B:** Scatter plot showing the correlation between longitudinal changes in SARA gait score and % of time spent in sedentary activities recorded from waist sensor in FRDA patients. The correlation is statistically significant (ρ= 0.517; p=0.03; Bonferroni uncorrected). CPM: counts per minute.
